# Intracranial pure yolk sac tumor in the anterior third ventricle of an adult: A case report

**DOI:** 10.3892/etm.2014.1945

**Published:** 2014-09-02

**Authors:** SUHONG ZHAO, GUANGRUI SHAO, WEIHUA GUO, XIUBIN CHEN, QINGWEI LIU

**Affiliations:** 1 Department of Radiology, The Second Hospital of Shandong University, Jinan, Shandong 250033, P.R. China; 2 Department of Radiology, Provincial Hospital of Shandong University, Jinan, Shandong 250021, P.R. China

**Keywords:** germ cell tumor, intracranial yolk sac tumor, anterior third ventricle, magnetic resonance imaging, radiotherapy

## Abstract

A 45-year-old female patient presented with symptoms of polydipsia and polyuria, menopause, headache, gait disturbance and deteriorated mental state. Brain magnetic resonance imaging (MRI) showed an irregular mass in the anterior third ventricle. The tumor was excised using a transfrontal approach from the anterior section of the third ventricle. The histological diagnosis was of an intracranial pure yolk sac tumor. The patient underwent radiotherapy and suffered no tumor recurrence one year after the surgery. Overall, when heterogeneous enhancement and an irregular mass with surrounding invasion and ventricular dilation are observed in the anterior third ventricle of an adult, a yolk sac tumor should be considered, and MRI may aid the differential diagnosis. A combination of surgical resection and radiotherapy is recommended for the yolk sac tumor.

## Introduction

Intracranial germ cell tumors are classified into two fundamental types: Germinomas and nongerminomatous germ cell tumors. Yolk sac tumor (YST), also known as endodermal sinus tumor, is a nongerminomatous germ cell tumor (1,2). Twelve percent of nongerminomatous germ cell tumors are mixed germ cell tumors with YST elements, and 2% are pure YSTs (3). Intracranial YST usually occurs in childhood or adolescence and is located in the pineal gland, suprasellar and posterior third ventricular region, and rarely in other locations (4). The present study describes a rare case of pure YST in the anterior third ventricle that infiltrated into the lateral ventricle in a middle-aged patient.

## Case report

**Figure 1:**
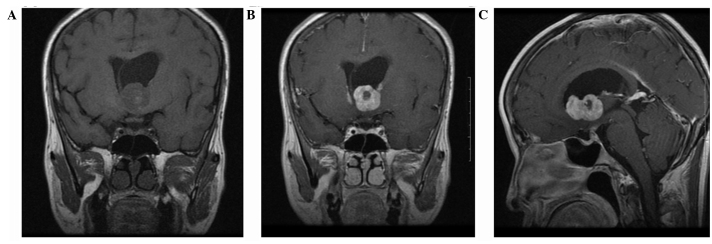
MRI observation of the solid mass in the anterior third ventricle. (A) Non-contrast-enhanced coronal T1-weighted MRI showing low signal intensity of the solid mass in the anterior third ventricle, which infiltrated into the lateral ventricle. (B) Contrast-enhanced coronal T1-weighted MRI and (C) contrast-enhanced sagittal T1-weighted MRI showing strong heterogenous enhancement with a non-enhanced hemorrhage. MRI, magnetic resonance imaging.

A 45-year-old female patient was admitted to the Second Hospital of Shandong University (Jinan, China) on October 31, 2012. The patient presented with symptoms of polydipsia and polyuria for 19 years, menopause for two years, headache for nine days, gait disturbance and deteriorated mental state, but with no evidence of visual field defects or papilledema. Brain magnetic resonance imaging (MRI) performed on November 1, 2012 demonstrated the presence of a mass in the anterior third ventricle with dimensions of 3.2×2.4×2.3 cm, appearing with low intensity on T1-weighted images, and hyperintensity on T2-weighted images. Strong gadolinium enhancement was present with the exception of at the hemorrhagic center. The tumor had spread to the left section of the lateral ventricles, and the third and lateral ventricles were dilated (Fig. 1). The neuroimaging diagnosis was of craniopharyngioma.

On November 12, 2012, the tumor was excised using a transfrontal approach from the anterior section of the third ventricle. The tumor was located in the anterior third ventricle, blocking the interventricular foramen, and partially invaded the lateral ventricle through the interventricular foramen. Immunohistochemical staining of α-fetoprotein (AFP) showed strong cytoplasmic staining. The histological diagnosis was of intracranial pure YST. Subsequently, the patient received whole-brain radiotherapy (30–36 Gy) and focal radiotherapy (45 Gy). All the procedures carried out in the present study were approved by the ethics committee of Shandong University (Jinan, China) and written and informed consent was obtained from the patient prior to participation. During the 12 months of follow-up, the majority of the symptoms disappeared with the exception of headache. MRI revealed chronic hemorrhage but no residual tumor.

## Discussion

Since radiological manifestations can be nonspecific, the preoperative diagnosis of a pure YST is challenging. In the present case, the mass was irregular and located in the anterior third ventricle, appearing as an area of low intensity on T1-weighted images and an area of hyperintensity on T2-weighted images. Strong gadolinium enhancement was present with the exception of at the hemorrhagic center. The preoperative diagnosis in the present case was of craniopharyngioma. Hemorrhage is not a characteristic radiological feature of craniopharyngioma (5). The differential diagnosis of YST in the anterior third ventricle includes craniopharyngiomas, germ cell tumors, gliomas, pituitary macroadenomas and meningiomas. Among these, germinomas are the most difficult to distinguish from YSTs. MRI and advanced MR techniques are useful in distinguishing between germinomas and YSTs. The solid components of germinomas may show homogeneous or heterogeneous enhancement, while YSTs show heterogeneous enhancement with hemorrhage and necrosis (6,7). Diffusion-weighted imaging (DWI) is reportedly helpful in differentiating germinoma germ cell tumors from YSTs. YSTs show facilitated diffusion with a high apparent diffusion coefficient value (8). The value of DWI and other functional MRI studies in the diagnosis of YSTs requires further study. Since there are no specific imaging features of YSTs, serum or cerebrospinal fluid markers are necessary for diagnosis. An increased serum AFP level may indicate the presence of a YST. The AFP level was significantly increased in the present case.

According to the classification of intracranial germ cell tumors described by Matsutani *et al* (3), YST belong to the poor prognosis group, for which a combination of surgical resection, chemotherapy and radiotherapy is recommended (9). In the present case, the patient received radiotherapy alone. One year after the surgery, no tumor recurrence was observed. However, the long-term effect remains uncertain and requires further investigation.

In conclusion, to the best of our knowledge, the present case is the first report of a pure YST in the anterior third ventricle of a middle-aged patient. Although the definitive diagnosis of intracranial YST depends on the pathologic examination (10), MRI may aid the differential diagnosis. When heterogeneous enhancement and an irregular mass with surrounding invasion and ventricular dilation are observed in the anterior third ventricle of an adult, clinicians should consider the possibility of a YST. A combination of surgical resection and radiotherapy is recommended.
